# Cyber-Physical System Integration of IoT Sensing and Machine Learning: A Cross-Domain Review of Decision Support and Control in Smart Buildings and Precision Agriculture

**DOI:** 10.3390/s26144435

**Published:** 2026-07-13

**Authors:** Panagiotis Christias, Mariana Mocanu

**Affiliations:** Faculty of Automatic Control and Computers, National University of Science and Technology POLITEHNICA Bucharest, 060042 Bucharest, Romania; mariana.mocanu@cs.pub.ro

**Keywords:** machine learning, decision support, control systems, cyber–physical systems, IoT, smart buildings, precision agriculture, profit-aware optimization, cross-domain analysis, closed-loop control, control-oriented machine learning

## Abstract

A new generation of smart buildings and precision agriculture is evolving through the integration of cyber–physical systems (CPS), which combine IoT sensors with machine learning (ML). As such, there is an implicit assumption made by researchers in most of these studies that the ML component represents the decision making mechanism within the overall system. Furthermore, most researchers do not articulate the full scope of the cyber–physical feedback loop linking prediction outputs, operational decisions based upon those predictions, actual actuation of the physical plant or farm operation, and subsequent performance evaluations. The outcome of this paper brings out transferable decision support patterns across domains such as the mechanisms which have proven to be effective in scenarios with low number or quality of data measurements. Specifically, we present a review for two CPS domains that benefit intensely from decision support: smart buildings and precision agriculture. We examined how sensing, data processing, ML, and control modules are combined in practice when creating decision support applications. This resulted in a review of the literature to identify architectural patterns, decision objectives, and feedback mechanisms in both domains. This combination insight paves the way for more flexible and more effective decision making applications compatible with different domains.

## 1. Introduction

### 1.1. IoT and CPS: Definitions and Scope

IoT is a vast network of “smart” objects—devices, sensors, etc.—equipped with electronics, software, and network connections, which allow them to monitor the environment around, gather information, and send it across the internet or another type of computer network. It creates a digital platform in the physical world, allowing remote monitoring and automatic data transfer without direct human interaction [1]. In general terms, IoT enables the sensing and communication components of today’s smart systems and forms the basis for many modern decision support processes.

On the other hand, CPS represent integrated closed-loop systems combining both algorithmic computation and physical processes in order to facilitate automated decision making and control [2,3]. These systems continuously collect data about the physical world using sensors, use intelligent computational tools such as ML algorithms for processing and decision making, and finally act upon the environment using control signals sent to actuators, thereby completing the sensor–decision–actuator feedback loop. While IoT focuses primarily on creating connectivity and gathering data from many distributed devices, CPS focus on utilizing that gathered data to automatically control physical processes in real time. IoT may be viewed as a foundation component or subset of CPS enabling connected devices to provide CPS with necessary data to make coordinated decisions based on that data [4]. One of the focal points of this review is that IoT-enabled CPS represent the backbone of smart buildings and precision agriculture applications where sensors transmit data to DSS and control algorithms to optimize operational performance of various physical infrastructures. At a macroscopic level, CPS can be thought of as a set of computing devices that exchange information amongst themselves and with their surroundings through sensors and actuators in a feedback loop arrangement [3,5,6,7]. The key characteristics of CPS include integration of sensor data into the virtual world of information, deterministic behavior, significant flexibility in adaptation to changing circumstances, and the ability to manipulate complex data sets [3,6,8,9,10].

### 1.2. Existing Related Work

Recent articles have explored the applications of ML and artificial intelligence (AI) in smart buildings, primarily on energy efficiency, including prediction and monitoring, as well as load forecasting, fault detection, and analytics-driven optimization [11,12,13,14,15]. They emphasize forecasting accuracy, control strategies, and algorithmic performance within the building domain. Typically, the reviews do not explicitly address automated control nor decision support frameworks but instead focus on categorizing methods used in terms of control strategies, energy savings, etc.

At the same time, there have been a number of articles addressing precision agriculture and smart irrigation focusing on IoT-based sensing technologies, ML prediction models, and automatic scheduling of irrigation. Similar to the reviews focused on smart buildings, many of them tend to focus on sensing technologies, learning paradigms, digital twins, and water usage efficiency enhancements and frame irrigation as an optimization problem powered by data within the agricultural domain. In most cases, although decision support systems (DSS) are referenced in the review papers, they were generally treated as application contexts, rather than as central methodological components. Moreover, economics-related decision criteria such as profitability, payback periods, or return on investments (ROIs) were either implied or only separately analyzed/evaluated in the case of precision agriculture [16,17,18,19,20,21]. Table 1 provides an example of differences in how typical articles reference DSS, economic objectives, and multi-domain synthesis.

**Figure 1:**
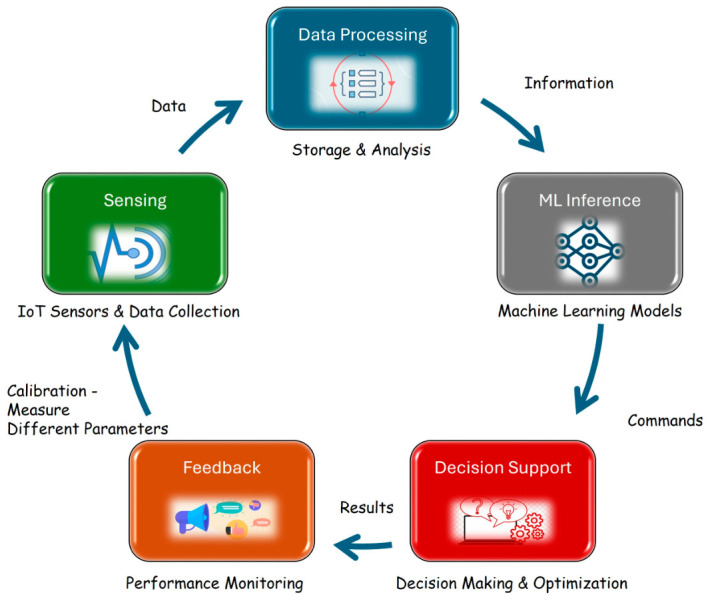
IoT CPS feedback loop with explicit separation between ML inference, decision support logic, and control actions (this study).

This work differs from existing research which mostly focuses on the algorithmic or application areas. The proposed review is an examination of IoT- and ML-enabled decision support and control. This examination is performed around the CPS feedback loop structure which includes sensing, data processing, ML inference, decision logic, actuation, and feedback [3,6,22]. Smart buildings and precision agriculture will be studied jointly as two economically and technologically interesting areas where decisions are directly correlated to control actions such as Heating Ventilation Air Conditioning (HVAC) set points, retrofit selections, and irrigation schedules [13,14,23,24]. An additional significant contribution is the incorporation of profit, ROI and payback-aware decision objectives along with energy and water efficiency. This allows for a cross-domain analysis which has been missing from most articles currently available [14,21,22,25]. Figure 1 depicts the CPS feedback loop approach to provide a common framework for comparison of the two domains.

Figure 1 shows the conceptual feedback loop of an IoT CPS utilizing ML-based decision support. Distributed sensors collect raw data that is processed and analyzed to produce ML inferences. These inferences then provide information to a decision making/optimization layer, and those decisions are implemented using physical actuators. Performance is perpetually evaluated as data is collected from each sensor. As can be seen by applications in smart buildings and precision agriculture, they share the same architectural framework, but each one uses application-specific objective functions, constraint sets, and actuators. To the best of the authors’ knowledge, this article attempted for the first time to examine smart buildings and precision agriculture as two application domains that are representative but also provide complementary applications.

Although the sensing–inferencing–acting loop shown in Figure 1 can be seen as similar to general CPS and control architectures, the key contribution of this study is in seeing this loop through a “decision support” lens. CPS models typically treat control logic as a predefined or embedded component. Compared to that, the separation above of ML and decision support and control actions into separate functional layers provides a variant conceptual model for decision making which concerns real-world applications.

Specifically, while it may be common to represent ML as an autonomous controller, the representation here treats ML simply as a generator of predictions, estimates or evaluations used by a higher-level decision support layer where operational objectives and constraints are determined together with tradeoffs.

Our concept also makes explicit the inclusion of multiple objective decision criteria (e.g., economic considerations such as cost, ROI and resource utilization). Usage of these types of criteria is rare in conventional CPS models, and when they are included, it is usually implicit. Therefore, the addition of explicit multiple-objective decision criteria will allow for unified analytical models applicable across multiple application areas. Unlike heavy learning control approaches (such as reinforcement learning), where models directly generate control actions, the perspective adopted in this work emphasizes architectures in which ML provides predictions and evaluations that support, but do not replace, a decision support layer responsible for selecting actions under certain constraints and objectives.

## 2. Methodology

A scoping review methodology is employed in this study. A scoping review is an ideal methodology for combining research areas with wide-ranging concepts, varied methodologies and rapidly changing domains. We explore ways to map existing evidence, determine typical designs, and identify research gaps. The primary goal of our scoping review is to analyze and synthesize the recent literature related to the topic. We will not seek to perform comparative analyses or develop a ranking system [26,27].

Given the nature of the methodologies discussed in this paper, this study’s objectives align closely with established scoping review frameworks. That is, while this study seeks to synthesize the relevant literature regarding decision support and control, it does this in a manner designed to facilitate interpretation rather than to include an exhaustive list of all available studies. The two application domains addressed—smart buildings and precision agriculture—share characteristics that are applicable to both. Specifically, both applications rely heavily on sensing provided via IoT technologies, utilize ML inference, and seek to feed decisions based upon actual measured results related to resource efficiency and economics [28].

### 2.1. Source Identification

Peer-reviewed journal publications, conference proceeding papers and authoritative articles dealing with the disciplines of IoT, ML, control systems, and Applied CPS comprise the source material used for this review. We included studies which focus on integration of data-driven inference with decision processes or mechanisms. Examples can be found in HVAC control, retrofit selection, irrigation scheduling, etc. The article databases from MDPI, Elsevier, Scopus, Science Direct and IEEE were used. We primarily focused on studies published from 2019 onwards, considering the evolution of IoT and ML in CPS, while selectively including earlier foundational works where necessary. Keywords used in the search process included: “IoT”, “machine learning”, “cyber physical systems”, “decision support systems”, “control”, “smart buildings”, and “precision agriculture”.

Instead of restricting this review to narrow definitions of algorithmic classification, potential sources were evaluated based on their ability to demonstrate how ML contributes functionally to the CPS feedback loop—that is, perception, prediction, decision logic, and actuation support.

### 2.2. Inclusion Criteria and Conceptual Boundaries

In order for a study to be included in this review, it had to meet one or more of the following criteria:

- The study combined data acquired via IoT-enabled devices with some kind of ML model or inference.
- The study supported decision processes or control actions through one or more decision support layers.
- The study focused primarily on smart buildings or precision agriculture.

Passive monitoring or visualization and predictive analytics alone that did not contribute to subsequent decision making or control activities were deemed outside the scope of this review—unless the study indicated linkage between predictive analytics and the design of control systems.

### 2.3. Literature Search and Selection

**Figure 2:**
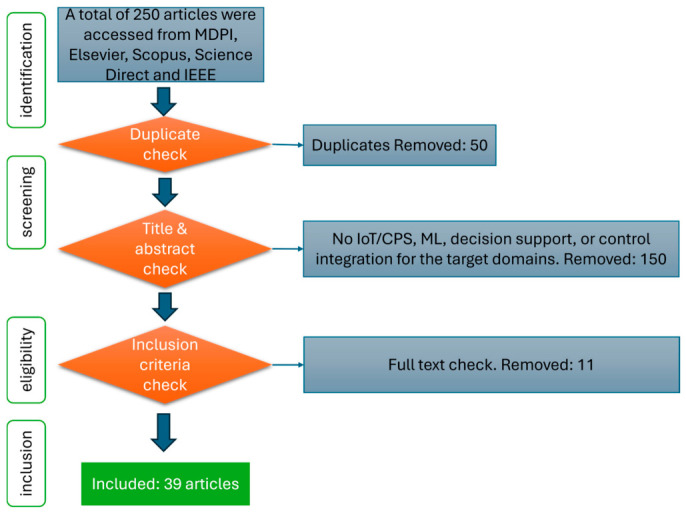
PRISMA-ScR flow diagram for study selection. The final qualitative synthesis included 39 studies after duplicate removal, title/abstract screening, and full-text eligibility assessment.

A structured scoping approach was applied to identify and evaluate the literature through conducting a scoping review, which utilized the PRISMA-ScR framework as a guide to ensure all steps of the study were transparent, reproducible and could be reported according to established guidelines. Using the database list discussed in Section 2.2 we executed a comprehensive search with the following terms: (((“smart building*” OR “building management”) OR (“precision agriculture” OR “smart farming” OR “smart irrigation” OR “agriculture 4.0”)) AND (IoT OR “Internet of Things” OR “cyber-physical” OR CPS) AND (“machine learning” OR “ML” OR “artificial intelligence” OR “AI”) AND (“control” OR “decision support”)). There were 250 total records identified. Following removal of duplicate records (50 duplicate records), we screened 200 titles and abstracts. At this stage, 150 of these were removed from consideration because they did not address the integration of IoT/CPS, ML, decision support, or control in the target domains. The remaining 50 were examined against our inclusion criteria outlined in Section 2.2. Following the examination of full-text versions of each article (50), we assessed them again for inclusion based on their relevance to the topics. We eliminated 11 articles at this point due to lack of closed-loop decision making components and/or lack of focus in one of the two target domains (smart buildings/agriculture). As a result, we identified a total of 39 articles that were ultimately used in the qualitative synthesis portion of this review. The flow diagram for the selection process is depicted in Figure 2. The final included studies are summarized in Tables S1 and S2 (Supplementary Material).

### 2.4. Synthesis Strategy

Qualitative analysis was employed to organize extracted data according to a CPS-centric taxonomy as presented in Tables S1 and S2 (Supplemental Material). It focused on the following:

- Where ML was inserted into the CPS architecture and what type of decision support structures existed within each study.
- Where and how control actions occurred.
- What the contexts were for evaluating effectiveness.

As is expected with qualitative analyses that incorporate a scoping review methodology, the resulting synthesis provides opportunities for cross-domain comparison, as well as for identifying challenges/considerations, to align with the intent of a scoping review.

## 3. Architecture of CPS

CPS may be thought of (from an engineering standpoint) as physical systems being operated in a rational manner with feedback mechanisms destined to monitor, control, or manipulate them. Thus, some conceptual foundations to CPS exist, dating back to the early days of developing feedback control systems during the Industrial Revolution when sensing, actuation and control logic were first integrated into physical processes.

Advances in computing technology over the last decades have greatly increased this system’s potential. Large scale networking, coupled with the ability to exchange information via the internet, has revolutionized how we act upon information exchange. Additionally, the widespread use of embedded processors, sensors, and wireless communications has made it easier to build continuous interactions between computer systems and the physical world [5,6].

**Figure 3:**
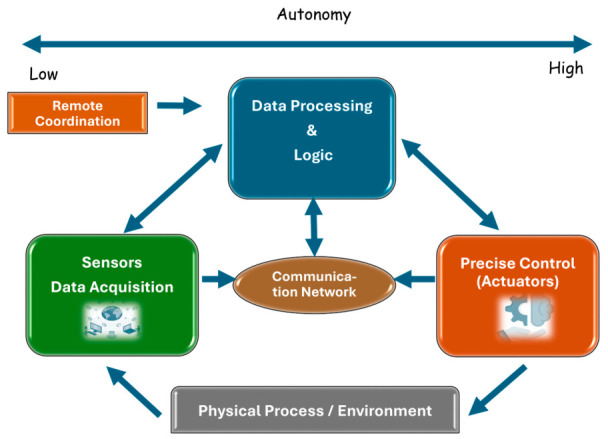
CPS capabilities schema.

Modern CPSs typically include a combination of five core capabilities (Figure 3):

1. Data processing.
2. Communication.
3. Precise control.
4. Remote coordination.
5. Varying levels of autonomy.

The scope of this perception—analysis—decision—actuating generalized model extends beyond the domains reviewed in this study. Recent research on other domains like IoT-enabled CPS architecture for aerospace smart manufacturing demonstrates an integration of multiple types of systems such as sensors, analytics, decision support software, and automation to execute integrated manufacturing, assembly, test and acceptance functions. Aerospace manufacturers use CPS technology to convert raw data into actionable business intelligence to enable them to optimize their process, identify anomalies in their process, utilize resources, and make long-term plans for future production. Although aerospace manufactures may be pursuing different goals than building owners or farmers, they all follow the same architectural paradigm as described previously. Sensors provide state information. Analytics/ML assist with decision making. Actions are executed based upon decisions made, and results are fed back into the decision loop. These observations provide even more evidence that the CPS feedback loop paradigm identified in this paper represents a generalizable architectural framework versus a domain-specific solution [29].

These architecture advances provide much of what is referred to as “digitalization” or “the digital transformation,” in the spectrum of engineered systems. In particular, CPS are essentially the technological base for IoT. Further development of data analysis techniques and ML are continuing to expand the capabilities of CPS by allowing systems to not only sense and react but also provide predictions and support adaptive decision processes [22].

Several technological trends will continue to drive the progression of CPS. These include advancements in low-cost but high-performance sensors, advancements in low-power embedded computing hardware, increased throughput and lower latency wireless communications, and advancements in energy storage and power management. All these factors lead to large-scale, safety-critical CPS which function as part of open, cloud-based frameworks and boost daily life activities and industrial operations. This architectural foundation is the basis of how IoT- and ML-based CPS are used to support decision making and control for smart buildings and precision agriculture and is presented in subsequent sections [4].

## 4. Decision Control and Support in Smart Buildings

Smart buildings represent an example of CPS in which sensing, computation, and actuation work together to create optimized building services including HVAC, lighting, and indoor environment quality. Reviews of recent research indicate that ML has been widely adopted to enhance predictive analytics, automation of control, and especially to reduce energy usage, while maintaining designated comfort levels.

### 4.1. Closed-Loop Operation in Smart Building CPS

**Figure 4:**
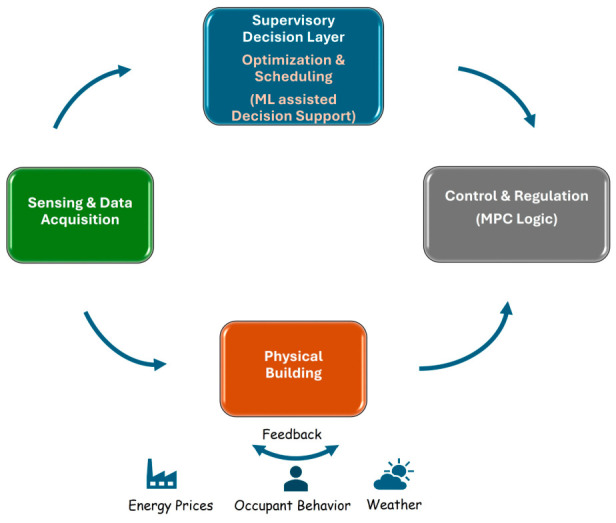
Closed loop in smart buildings.

As described above, smart buildings are increasingly conceptualized as CPS that operate in closed-loop mode. The physical building operations are constantly monitored, analyzed, and controlled through the application of computational decision tools and control strategies. Unlike many existing building management systems, which often rely on predetermined rules or static schedules, a closed-loop CPS smart building allows the system’s behavior to constantly respond to changes in its internal operating environment and external environmental conditions (Figure 4) [30].

#### 4.1.1. Sensing Stage

Sensing constitutes the initial phase of the control cycle in a closed loop building CPS. Distributed sensors embedded across the building collect information in real time about physical variables associated with the building such as indoor temperature, humidity, air quality, occupancy levels, energy consumption, and equipment states. Through this process, CPS provide an ongoing understanding of the physical state of the building and its current operating context which is used to make subsequent inferences and decisions [31].

#### 4.1.2. Decision Stage

The decision making layer converts sensed data into information for evaluation. This layer includes a variety of functions including state estimation, prediction, and optimization. They are often performed using ML models. Instead of taking autonomous action based solely upon their own analysis and recommendations, most ML models function as decision support tools that generate predictions, estimated values for unknown parameters, or evaluated options which are then fed to higher-level supervisory control logic. Therefore, the decision process depends on sensor feedback as well as constraints and goals (such as energy efficiency, occupant comfort, etc.) [11,12,15,31,32].

#### 4.1.3. Actuation Phase

The final step in the loop involves converting decisions into physical actions through the utilization of actuators such as setting HVAC set points, adjusting valve positions, varying fan speed, etc. Once implemented, actuation results in direct alteration on how the building behaves. Subsequently, these changes affect future sensor readings, thereby enabling cyclical feedback and adaptation in terms of performance. A key benefit of closed-loop operation is that control decisions are inherently evaluated by their effects on the building’s behavior. This way, the system gains the ability to adjust over errors, disturbances and variations due to occupants’ interactions and weather conditions over time [33,34].

Finally, closed-loop CPS operation in smart buildings occurs across both time and space domains. Fast regulation loops (i.e., temperature or pressure regulation) coexist with slower supervisory loops (i.e., energy management, scheduling, and retrofit decisions). Therefore, the decision support layer acts as the coordination point between sensing and actuation instead of being a standalone predictive element [14,20,35].

### 4.2. Smart Building IoT Data Streams and Closed-Loop Systems

The participation of various types of devices, which are interconnected via the internet, enables smart buildings to create an environment with continuous, real-time data collection and utilization. Most systematic reviews regarding smart buildings have identified the use of different types of sensors with purpose to monitor environmental conditions, energy usage, occupancy, etc. In addition, many reviews indicate that the integration of these diverse sources of sensor data across subsystems (HVAC, lighting, energy management) is valuable if fully utilized by decision support [35].

While integrating these subsystems facilitates decisions that consider system-wide goals, this creates new puzzles coming from data incompatibility, synchronization when collecting and processing data, and increased system complexity. All these challenges affect the reliability of decision support [30,36].

In addition to identifying data integration as a key challenge when designing decision support in smart buildings, complementary literature has demonstrated that energy efficiency and control are not simply model-based solutions. In fact, deployment realities and limitations (integration complexity, cybersecurity/privacy concerns, data quality issues, and user acceptance) significantly affect the ability of decision support to effectively optimize energy utilization and control. These limitations impact which data can be sensed, at what frequency, and how confident one should be when utilizing sensed data in real-time decision logic [35,36,37].

Generally, the relationship between sensing and control processes is crucial as far as the decision mechanisms are concerned. But when sensor readings are noisy, inconsistent or missing, this has a negative impact on predictive and optimization models. Decision support designs must therefore exhibit resiliency against uncertainty and partial observability. Studies point out that powerful decision support reside on aggregated data or proxy parameters rather than discrete measurements of the building physical states. It is evident that bridging sensing and actions with effective inference layers in between matters more than the accuracy of predictions alone [33,36,37].

Lately, the trend in smart buildings is moving away from plain monitoring toward effective closed-loop decision support. Research around IoT considers smart building groups of discrete subsystems (like HVAC, energy monitors and indoor environmental quality). Sensor networks and data processing play the role of coordinator in trying to achieve control objectives.

At the same time, other scoping reviews indicate that the optimization of energy consumption is influenced by a variety of deployment considerations beyond pure modeling and prediction tasks. These include system integration complexities, limitations imposed by cybersecurity, privacy requirements and end-user acceptance. Overall, these findings suggest a closer look at where and how ML components are integrated into the building control stack. This is explored further in the next section [35,36,37].

### 4.3. ML Inside the Building Control Stack

Smart buildings articles usually do not describe ML as an end goal but instead as an aid in closed-loop decision making. There are generally three main aspects that reviews have categorized on how ML can be used in smart buildings, which ultimately drive operational choices [11,15]:

1. Forecasting/Predictions (for example: energy consumption, indoor temperature).
2. Operational optimization and automated control.
3. Maintenance related decision making (for example: fault detection, diagnostics).

A separate body of research focuses on buildings energy management using control-oriented frameworks, especially model predictive control (MPC). MPC emphasizes how predictive models and optimization can be used to determine what control actions should be taken within explicit constraints. In these types of frameworks, ML is typically used to aid predictive models, surrogates, or multiple-objective evaluation. However, the decision objectives and constraints are still placed at the supervisory control level versus being determined by the learning algorithm itself. This separation provides additional evidence that ML supports decision making while control authority is maintained by closed loops linking decision making with actuators and sensors [38].

A common thread throughout the smart buildings literature is that predictive control and optimization will only be effective if they are tightly coupled with building dynamics and the physical operating boundaries of a building. A few reviews emphasize that forecasts of energy demand, temperature, or occupancy are not useful in isolation and that their value depends on how they are incorporated into DSS, which honor networks delays and limits regarding safety, comfort and actuator capacity [34,38,39].

Predictive models are exploited for input in optimization or planning problems and not for autonomous actions. Relevant articles reveal that major concerns are operational objectives and ML components target high forecast accuracy or tradeoff analysis. It is concluded that in real-world case studies, decisions which satisfy constraints are more preferable than data-driven actions [38,39].

Real-life issues such as unexpected behavior of occupants, limited sensors coverage and lack of automation infrastructure presented by several studies question the effectiveness of predictive control. That is why hybrid control solutions are tested where ML assists during evaluation or prediction and higher structures are authorized to select which actions to take and when [14,39].

After discussing these findings, we propose a categorization of ML roles in decision processes to describe how ML models can fit in closed loops of smart building CPS.

1. Estimating the Perception State.
  ML methods can be executed to estimate information for unknown or less-observed states (for example: occupancy patterns, comfort levels or anomalies) using sensor readings. Those estimations are considered “within scope” not when they assist monitoring but if there is follow-up control logic.
2. Prediction.
  Energy consumption and indoor temperature oscillations constitute two prime examples of ML applications to predict building performance. Such predictions can guide higher levels of control and optimization processes.
3. Control decisions.
  Ultimately, ML may be responsible for selecting actions which will improve building operation and energy consumption. ML has different roles in various implementations of smart building control applications. In most of them, it integrates with control frameworks [11]. One of the most common ways to implement this is by using the output of an ML model to inform the inputs into a control framework (i.e., MPC) and then determining control actions based on the outputs of the MPC (which include both constraints and objectives, i.e., bounds on temperature, humidity and energy consumption) [38,40]. In other implementations, ML models contribute to action evaluation and selection by estimating the outcomes of alternative control strategies and then enabling decision support components to choose among candidate actions. Hybrid approaches are also reported, where rule-based logic, heuristics, or domain knowledge are combined with ML predictions to determine control actions under uncertainty or limited data availability [41].
4. Measurements and Direct Actuation.
  More recently, learning-based control approaches—such as reinforcement learning—have been explored, where ML models directly learn policies that map system states to control actions. However, such approaches remain relatively limited in real-world deployments due to issues related to safety, interpretability, and constraint handling. As a result, the dominant paradigm in CPS applications remains one in which ML supports, rather than replaces, structured decision and control logic. Therefore, the contribution of ML to control decisions should be understood not as autonomous action generation, but as enhancing the decision space from which feasible and constrained control actions are selected [39,42]. This distinction clarifies that ML contributions to control in CPS are primarily mediated through decision support layers rather than through direct actuation policies, reinforcing the decision-centric interpretation adopted throughout this review.
  As emphasized by this review, ML is mainly exploited as a decision support sublayer in most practical CPS implementations. Some researchers, though, focused on learning-based methods like reinforcement learning to train models towards optimal control policies from state monitoring to actions with no reliance upon predefined optimization or rule-based decision layers. Recent experimental work has exhibited the potential of learning-based control methodologies for enhanced smart building performance. It is also reported by the same researchers that there are important practical challenges. To date, most evaluations of deep/reinforcement learning methodologies have taken place in simulated environments as opposed to being implemented at the full-scale level of real-world systems. Additionally, the performance of the methodologies has been found to be dependent upon both the design of the algorithms used and the operational characteristics of the systems with which they are employed. Value-based approaches like deep q-networks have shown issues in certain operational regimes and require alternative formulations or architectures to provide a dependable level of control performance [42,43].

Judging from the preceding paragraphs, ML usefulness lies in its ability to embed into a closed-loop decision support system while preserving realistic deployment constraints. In Section 5, similar insertion points and integrations difficulties are studied in the context of precision agriculture, highlighting this way as the orientation for better decisions.

### 4.4. Decisions for Optimizations Regarding Energy, Comfort and Economic Viability

Although reducing energy usage is the primary consideration when optimizing smart buildings via use of ML, multiple reviewers have pointed out that decision making processes and controls will need to meet three types of constraints: those related to the occupants’ comfort, those related to the systems reliability and operability, and those related to economics in order for the optimization decision support process to be successful. Thus, smart building decision support is an inherently multi-objective operation [11,38].

Smart building CPS have started to include economics as part of the decision support process. There has been increased incorporation of cost-related objectives (such as energy cost, ROI, lifecycle cost, etc.) into the optimization control frameworks including MPC and planning-based scheduling. Decision formulation can balance both technical constraints and economic objectives, thus revealing a direct relationship between performance and cost [13,38,39]. While there are numerous economic metrics available to evaluate system performance (cost savings, payback period after deployment, etc.), they do not often appear in real-time decision logic as first class objects—this highlights a disconnect between how economic models are applied for evaluation purposes vs. decision support mechanisms [13,14,35,36,37,44].

Authors also found that while there are some technical issues related to deployment and long-term verification of the effectiveness of the technology, the technical performance of the solutions has been proven inadequate to encourage widespread adoption [13]. The results of these studies are supported by several IoT-focused systematic reviews that identified additional barriers to widespread adoption of smart building solutions. These include the following:

- High upfront costs associated with implementing new smart building systems.
- Complexities in integrating existing building systems with newer smart building systems.
- Security and privacy risks associated with collecting and analyzing data from smart sensors and other devices in a building.
- Dilemmas as to whether smart building solutions provide sufficient economic returns to justify their costs.

Therefore, instead of simply considering the potential financial benefit of implementing new smart building systems, decision makers need to consider the potential business value and life cycle costs associated with smart building solutions during the decision making process itself [13,14,35].

Additional scoping studies on data-driven building technologies indicate that energy optimization should be considered a system-wide decision problem. That is, technical objectives (such as energy efficiency and greenhouse gas emission reductions) as well as economic objectives (such as cost savings and justifying investments in energy saving measures) need to be integrated into the same decision framework. This further emphasizes the role of decision support in facilitating tradeoffs between competing objectives, rather than finding the optimal solution to a single objective [13,14,44].

Within this context, decision support in smart buildings can be associated with utility-based and multicriteria frameworks. In such cases, control strategies are selected based upon their overall impact on three dimensions of sustainability: energy performance, occupant comfort, and financial viability. As noted above, this represents a direct alignment with the decision-centric application of ML that was described in paragraph 4.3. It also presents an opportunity for comparison with how ML algorithms are applied to make decisions on resource allocation in precision agriculture applications, which will be discussed in Section 5 [44].

### 4.5. Retrofit Decision Support Under Uncertainty

Smart building decision support also includes retrofit planning, which involves selecting the best from multiple alternatives and making decisions regarding retrofit options under uncertainty, using little data, and at considerable costs. Studies have shown several times that detailed or extensive sensor data may be unaffordable or unavailable for many commercial properties, so decision strategies are encouraged to use any available information (both complete and incomplete) when possible and provide the decision maker with a plausible option [45].

As a general model for decision support concerning retrofit selections, one can describe this situation as an expected utility decision process. This means that various retrofit choices will be compared against each other according to their probability of achieving a better energy performance level than currently exists and the potential financial outcome of the selected retrofit. The use of probabilistic reasoning (such as Bayes’ theorem) allows for the expression of the uncertainties related to future energy levels, whereas utility functions allow for the integration of the economic benefits and payback time as specific decision criteria. Additionally, fuzzy modeling can be used to make the assignment of utilities less arbitrary and to assign weights to transitions between different energy performance levels based upon the degree of improvement (i.e., small, medium, significant) [46].

This approach demonstrates how decision support in smart buildings expands beyond predictive capabilities by combining probabilistic inference, fuzzy logic, and economic assessment into a single analytical framework. Most importantly, these types of decision support models are compatible with practical realities existing on deployment. They address issues including poorly populated databases, variable characteristics of buildings, and cost-sensitive restrictions. Assessing the economic feasibility and uncertainty factors associated with retrofit decisions will remain a significant issue, though it is not universally considered as part of scalable deployments in real-world environments [13,37].

### 4.6. Scheduling and High-Level Decision Support

Decision support that incorporates scheduling and high-level planning for buildings has been an area of interest in recent years. In addition, there have been significant advancements in AI planning related to building operations and energy management. The primary focus of this type of research was on achieving cost-effective outcomes while ensuring that decisions are coordinated. As discussed in other sections of the review, it appears that decision support is becoming more prevalent at all levels, including low-level HVAC control, high-level scheduling/planning, and at the same time is driven by economic vectors and multiple objective functions [39,47].

### 4.7. Summary

The literature reviewed indicates there are many recurring trends associated with smart buildings:

- The CPS loop is becoming more dependent on data; however the quality of decisions heavily relies on how well models are deployed and integrated, rather than simply on their accuracy [14,36].
- Economic feasibility is mentioned repeatedly (cost, ROI) as an important factor. Nevertheless, it is not consistently incorporated directly into the logic for making decisions as much as other objectives (energy use, comfort, etc.) [13,14].
- Traditional approaches to controlling buildings (MPC and planning) continue to serve as the primary framework for providing decision support at the operational level. Additionally, ML is being used extensively as a supplemental tool for improving these traditional approaches [38,39].

The trends above suggest the potential for cross-domain comparisons with precision agriculture as described in Section 5, where similar types of decision support and actuation under uncertainty exist. Having said that, such cross-domain comparisons are subject to a variety of constraints (e.g., field variability and limited connectivity).

## 5. Decision Support and Control in Precision Agriculture

Precision agriculture can be viewed as a series of CPS applications that facilitate the use of data-driven approaches destined for better decisions on resource management. Sensors are used to gather information, ML algorithms to interpret data and designate possible courses of action, and actuators to implement those decisions form a closed-loop approach to resource allocation. The primary goal of these systems is to improve yields while minimizing resources waste. Recent studies have described the typical components of an agricultural CPS. Most commonly, they include sensing to collect data, ML to infer what the collected data indicates, and then passing on the inferred information to guide actuators towards actions within the physical world (e.g., irrigation at specific rates). In addition to the closed-loop character of these systems, there are also additional layers that exist inside these systems [20].

### 5.1. CPS Feedback Loops in Agricultural Systems

In contrast to traditional schedule-oriented farming practices, modern agricultural methods are utilized by closed-loop CPS that maintain continuous feedback between the physical environment (e.g., soil, crops, microclimate) and computational decision logic. Recent articles characterize agricultural CPS as feedback loops where data collected from the field under changing environmental conditions are continuously processed, analyzed and used to inform management actions [20,25].

**Figure 5:**
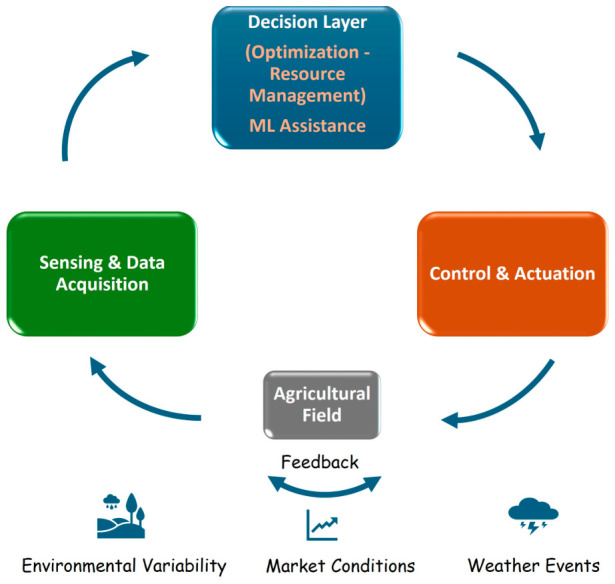
Closed loop in precision agriculture.

These systems usually consist of three layers (Figure 5):

#### 5.1.1. Sensing Layer

Dispersed sensor groups absorb information from the physical world that can be inserted into the computational decision logic. Common examples include soil moisture sensors, weather stations, and satellite/aerial images [48].

#### 5.1.2. Decision Logic Layer

Once information is received from sensors, it is enhanced with knowledge regarding best practices, models, and goals of operation. This layer utilizes ML algorithms to evaluate all available information and generate actionable recommendations for farmers. Numerous articles have indicated that ML enables the decision logic layer to utilize states (i.e., current condition), forecasts (predictions about future conditions) or alternatives (i.e., comparing two or more different scenarios) to assist in generating proposed actions. Apart from that, it is emphasized that decision logic must consider factors like uncertainty, field heterogeneity, and resource limitations when producing recommended actions [21,25].

#### 5.1.3. Actuation Layer

This layer concerns physical implementations related to decisions formed earlier. These may include irrigation control or time scheduling for field activities. Actuators are critical because they can interact directly with the physical world, thereby allowing for continuous monitoring and adapting throughout each growing season [48]. Like smart buildings, value of intelligent agricultural systems is not solely in sensing or predicting, but in the ability to convey predictions to control actions under uncertain conditions. For this reason, precision agriculture is considered a problem that deals with both decision support and control and does not simply collect and analyze data [20,25].

### 5.2. IoT Data Sources and System Integration

The involvement of IoT sensing technologies has been emphasized in recent systematic and scoping reviews as critical to the future success of precision agriculture. Use of multiple types of sensing technologies including soil moisture, temperature and humidity sensors, weather station data, and proximal and remote sensing allow for real-time monitoring of agricultural fields in space and time. Hence, since they enable the collection of data, sensor data supplied to an agricultural control systems platform represents the “perceptual” layer upon which decisions occur [48]. In contrast to buildings, where many sensors are densely connected via a network, many agricultural CPS are deployed in areas with limited access to trustworthy communications infrastructure and power. As a result, the reliability of individual sensors, their connectivity, and data heterogeneity in rural areas directly impact data quality, latency and, overall, the potential of closed-loop operation [12].

Processes in agricultural DSS also reflect operational constraints and challenges. While DSS may collect a wide range of data from multiple sources, articles have shown that they frequently employ aggregation techniques, inferential or proxy analysis, to impute missing and noisy sensor data [48].

It appears that IoT precision agriculture is handled as a system integration challenge, where sensing, communications, and analytics actively participate in decision support design. This distinguishes agricultural CPS from their buildings counterparts and raises the need for decision support which accounts for uncertainty, field and crop diversity, and infrastructural limitations [12,20,23,49].

### 5.3. ML Insertion Points

The literature across all reviewed studies identifies a consistent trend for the use of ML techniques as supportive elements or subcomponents within closed-loop agricultural CPS. The key, however, is that ML is generally not identified as an autonomous decision element. Instead, it provides improved input to perception, forecasting and evaluation functions in pursuit to better decisions. The decision control is still ultimately determined by agronomic objectives, resource constraints and operational feasibility [16,25].

The integration of ML into the closed-loop CPS can occur through identifiable insertion points, each one focused on a particular type of decision. While researchers highlight how efficiently ML algorithms predict certain types of data, few have investigated how the results of those predictions are converted from an analytical output to an actual control action or enable the user to understand how analytics relate to operational decisions [19].

One common thread throughout the literature is that predictive models are only useful if they support constrained decision processes. For example, forecasts of evapotranspiration, irrigation demand, or the immediate responses of crops to changing conditions must be viewed relative to factors such as field-to-field variation in soil and weather uncertainty, as well as limitations regarding access to water resources and operations. Thus, similarly to predictive analytics in smart buildings, predictive analytics in agriculture generally serves as input to decision support tools rather than as a generator of direct actions. To facilitate clear organization in this review around the involvement of ML in precision agriculture closed-loop CPS, we identify three layers of decision functions aligned with those closed-loop architectures described in Section 5.1 and Section 5.2:

1. State Inference.
  ML is utilized to convert diverse forms of sensor measurements collected across fields into descriptions of the current state of soils or crops, including metrics such as soil moisture deficit, crop stress indicators or spatial variation patterns. We consider only those inference tasks to be “within scope” when they provide inputs to downstream decision support or control logic rather when they serve descriptive monitoring activities.
2. Prediction.
  As mentioned earlier, ML models are commonly applied to predict quantities such as evapotranspiration, irrigation demands or short-term yields responses under alternating environmental conditions. The primary use of these predictions is to provide expectations regarding future behaviors of the system so that decision makers may implement scheduling and optimization.
3. Decision Support and Control.
  Ultimately, ML output is used to provide actionable recommendations for farmers mostly pertaining to irrigation scheduling, variable rate application, or timing field operations. Although ML provides inputs to decision processes in most of the systems studied, it does not replace explicit decision logic. Rather, it usually operates either within or alongside rule-based or hybrid decision frameworks and contributes to determining which actions should be selected.
4. From Sensing to Actions.
  While most of the literature on precision agriculture views ML as a tool for making irrigation decisions, there are some studies that view learning-based architectures as potential ways to map crop/soil sensor data into irrigation commands. There are examples where deep reinforcement learning has been applied to produce irrigation prescriptions based on daily readings from various sensors. The researchers claimed that such approaches were better than traditional irrigation rules. Other studies observed good results after applying reinforcement learning irrigation control as a fully closed-loop irrigation control policy [50,51,52,53,54]. However, evidence spans simulations, specially constructed experiment environments and greenhouse control. Tests are conducted using crop simulation models (e.g., APSIM/DSSAT) instead of deployed in open-field agricultural operations.
  Moreover, the performance of learning-based controllers seems to rely on the definition of the control problem along with the characteristics of the physical environment. Finally, contributors note that additional measures are needed to provide assurance that the learned control policy functions reliably. Examples include introducing safety/fallbacks into field-based reinforcement learning systems that would inhibit undesirable irrigation actions.

The layering described above presents another important aspect of both smart buildings and precision agriculture applications: regardless of whether a ML model produces accurate predictions, its practical contributions are best measured by the degree of integration into a closed-loop decision support structure, operating under uncertain conditions. This interpretation facilitates cross-domain synthesis as described in Section 6.

### 5.4. Decision Objectives: Water Efficiency, Yield and Economic Sustainability

Agricultural decision support is inherently multi-objective because it must consider three major goals: water conservation, crop yield, and financial viability. All irrigation and resource allocation decisions in precision agriculture are viewed as tradeoffs among agronomic performance, resource conservation, and financial constraints. This is especially true in cases with climate variability or severe water restrictions [16,21,25].

As reviewed above, irrigation decisions must balance biophysical constraints (e.g., soil properties, crop water requirements, etc.) with operational and economic factors (i.e., water availability, energy costs, etc.). Therefore, DSS for agricultural production extend well beyond identifying optimal water usage strategies or maximizing yields individually and aim to identify solutions which are both operationally effective and economically viable.

Economic factors are generally seen as key to making decisions in precision agriculture, particularly relating to profitability, input costs and yield maximization, but are often less formally defined in terms of decision support mechanisms compared to smart building systems [17,20,21]. Many reports describe economic considerations, but this is stated indirectly as in reducing water and energy usage [17,25,55].

Some DSS consider the cost implications when developing irrigation or resource allocation recommendations but most commonly rely upon heuristics or rule-based methodologies and not upon well-structured optimization frameworks [17,25]. Therefore, it is possible to leverage agricultural CPS with the inclusion of explicit cost functions, economic constraints into decision support mechanisms to enable long-term sustainability, and more systematic and transparent tradeoffs between agronomic performance and financial outcomes.

Therefore, from the CPS and decision making viewpoints, the challenge is to optimize multiple criteria under uncertainty where water consumption, crop responses to water application, and economic impacts are traded off against one another rather than being optimized individually.

### 5.5. Irrigation Decision Support Under Uncertainty

Decision support for irrigation under uncertain environmental conditions is another vast application of precision agriculture. In these scenarios, agronomists must combine agronomic knowledge (such as how crops respond to water) with limited, variable and often noisy measurements recorded at different points in time across the farm. Systemic reviews identify decision support for irrigation under variable and uncertain environmental conditions as one of the most common applications of agricultural CPS based on difficulties associated with collecting a comprehensive set of detailed measurements from all areas of the farm, the significant impact that weather variability has on crop water demand, as well as differences in soils responses to water reception [16,19,56].

The majority of reported systems utilize some form of rule-based reasoning (such as the Water Stress Index from FAO 56, or simple threshold-based heuristics) in conjunction with ML inference techniques or decision trees to provide irrigation recommendations. The hybrid formulation of these systems exploits data techniques to estimate current field conditions and make predictions about behavior in the short term while agronomical rules apply [56].

In many cases, fuzzy and multicriteria decision support models are also utilized to satisfy antagonistic agronomic factors like applied water, crop response and financial cost when there is no precise and affordable process model available to represent those relationships. Authors of recent reviews have noted that this approach is well suited to applications in agriculture, where uncertainty, missing data, and expert opinion characterize most decision processes [46].

Considering the decision support orientation of the aforementioned examples and their associated decision support patterns, we observe an analogy to the smart buildings paradigms previously discussed. Specifically, both domains require effective control under uncertainty, effective use of incomplete or aggregated information, and integration of technical performance metrics with cost effectiveness under a unified decision logic umbrella. This similarity reinforces the notion that irrigation decision support should not be viewed solely as a prediction problem, but also as a decision support problem, giving us arguments for the cross-domain synthesis described in Section 6 [14,25].

### 5.6. Control and Action in Agricultural CPS

In addition to providing recommendations, current research emphasizes irrigation scheduling and control as the final actuated component of an agricultural decision support pipeline. Thus, as ML is used to make decisions for a CPS, those decisions can be converted to specific physical actions (such as when to irrigate; which valves to open/close; how much water/inputs to apply), wiring this way the loop from sensors to action. Effective agricultural CPS exhibit quality predictive models as well as a high degree of integration with control elements (with irrigation systems hardware, network communications, and farm management systems). Additionally, since all environmental parameters are constantly changing, it is necessary for actuation algorithms to keep up under uncertainty and operate within engineering bounds related to limited water supply, energy, budget and resources [18].

As in smart building applications, the literature has repeatedly demonstrated that control performance occurs at the system level and not necessarily through individual model components. Therefore, the success of ML-enabled irrigation scheduling will depend on successful integration of decision logic, control execution, and feedback. This idea further supports the notion of precision agriculture as a closed-loop CPS versus a collection of standalone analytical tools [20,48].

### 5.7. Synthesis and Emerging Patterns

The synthesized results from the review process illustrate a number of common trends across decision support in precision agriculture. First, there is a rising trend towards closed loop sensing, decision support, and actuation in precision agriculture via CPS. On the contrary, many of these systems lack explicit description of the decision logic used by models. While some contributions concentrate primarily on data acquisition or predictive accuracy of model inputs, the mechanisms for translating input data into actual control actions are either implied or implemented using heuristic methods [25].

ML techniques have been predominantly used to predict water consumption, crop yield, etc. There appears to be little consistency in integrating ML output into a systematic and well-defined decision support framework. It is worth noting that the use of ML is also common in smart building control applications, where the application of constrained optimization and supervisory control architectures are far more developed [55].

Finally, although economic and resource efficiency goals are commonly stated in the context of agricultural decision support, they are usually treated implicitly as opposed to being formally formulated in the decision support models. The motivation for developing profit-aware and cost-constrained decision support for agricultural applications is addressed in Section 6 [57].

## 6. Transferable Decision Support Patterns Across Domains

In earlier sections, we used smart buildings and precision agriculture to illustrate decision support and control in CPS. While both CPS domains have application differences, a comparative examination of the two will reveal similarities in terms of structure, as well as potential areas for additional research that are less apparent in articles focused on each domain individually.

### 6.1. Similar Structural Pattern of CPS Decision Support Across Domains

Both domains’ DSS implement a structurally similar CPS feedback loop: Sensing → Inference/Prediction → Decision Logic → Actuation → Feedback. The smart building loop is commonly implemented via HVAC control, retrofit planning, or operation scheduling. The precision agriculture loop is commonly implemented via irrigation scheduling and resource management. The roles of ML in each domain were also consistent, to enhance perception and make predictions, while ultimate decision logic is responsible for determining what type of and when an action should be executed. Therefore, these studies confirm that CPS decision support is control-centric rather than algorithm-centric as outlined by many recent CPS and IoT studies [3,6,7,22].

### 6.2. ML Insertion Points—Common Ground, Different Focus

**Figure 6:**
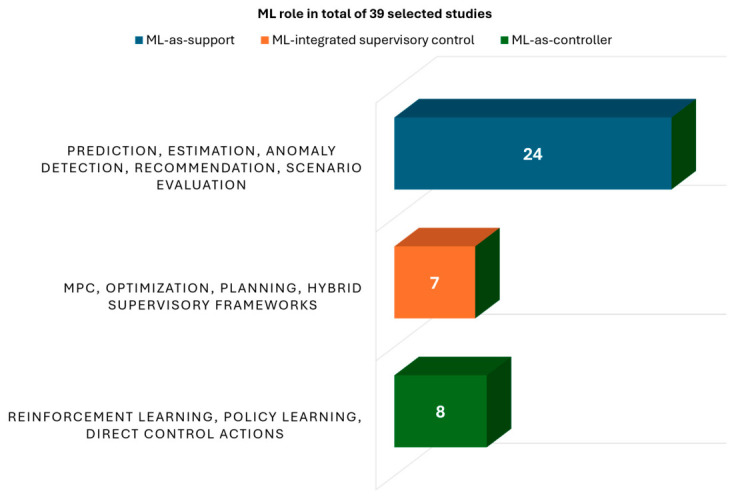
Taxonomy of ML role in the 39 included studies.

Smart buildings and precision agriculture use ML at similar points in the CPS loop. However, their focus differs. Smart buildings primarily use ML to forecast and optimize building operation via control structures like model predictive control or planning-based scheduling. Precision agriculture uses ML in a different way: to estimate states of variables that cannot be measured directly and to recommend actions. In most cases, data is an issue. There may be noise, delays or missing information due to abnormal sensor measurements. Agricultural CPS often combine ML with other forms of reasoning like agronomy rules, heuristics and fuzzy logic to account for changes in weather, soil moisture, temperature, etc. It is important to note that this difference represents constraints of the domains and not an indicator of a maturity lag in ML application [13,14,18,21,25,39]. To create an even clearer understanding of what role ML will play, Table S2 illustrates a hierarchical structure of the taxonomy created from all 39 of the selected articles (Table S1) with their coded categories. In this table, it is apparent that decision support (24/39 or 61.5%) is by far the largest category of how researchers have employed ML. Following decision support was the use of ML embedded into supervisory/hybrid control systems (7/39 or 18%), whereas less frequently, researchers have investigated whether ML could act directly as a controller (8/39 or 20.5%). The taxonomy of ML insertion points is formed and presented in Figure 6.

### 6.3. Economic Goals Among Decisions Objectives

A notable parameter in these domains is the form of integrating financial targets into decision tools. In smart buildings research, this is reflected in reductions in energy consumption and sometimes tied to ROI and payback criteria, especially when it comes to retrofit planning or deployment evaluation.

In agricultural DSS, there is a horizontal requirement of economic sustainability (profitability, reduced costs, stable yields), but economic goals are generally treated indirectly without being incorporated directly into decision making algorithms. As a result, economic factors are almost always considered after decisions have been made, and rarely do they influence which actions will be selected.

These differences suggest opportunities for transferring methods from one domain to another. Profit or utility-based formulas (common in building retrofit planning) could aid in developing decision models with more transparency in agriculture. The ability of agriculture-related decision support to handle uncertainty and low-quality data could improve decision integrity in the buildings’ environments [13,23,24].

### 6.4. Applicability

An important aspect of the transferable characteristics of CPS-based DSS is demonstrated by decision making processes where a given decision framework may be transferred from one domain to another. For instance, there is potential for a ROI-driven DSS for retrofitting in smart buildings to be applicable to the decision making process for managing irrigation in precision farming.

Decision makers in building retrofits typically compare several different options based upon anticipated energy efficiency, installation cost, maintenance needs, pay-back time, and total ROI. There are research examples which demonstrate that the best technical choice is not always the most likely to be implemented, since some form of justification for the cost of deploying is required to fund such solutions. Therefore, retrofit decisions are generally made using multicriteria decision methods that include a combination of both technical and economic aspects into a single set of solutions [13,14,15,44].

Similarly, there are numerous irrigation DSS used within precision agriculture that aim to optimize water usage or crop yields. However, the economic implications of such optimizations are rarely explicitly addressed [17,25,55]. By developing a ROI-based DSS on irrigation utilizing concepts analogous to those applied in building retrofit planning, irrigation decisions could be optimized with respect to anticipated crop yield improvements, water usage, irrigation costs, energy usage and anticipated returns. Instead of simply choosing an irrigation strategy based solely on agronomic metrics, an irrigation DSS could assess the anticipated value associated with each of the possible irrigation strategies identified by the ML algorithm and select the strategy providing the greatest overall economic benefits under resource limitations.

As an example, assume that an ML model predicts two different irrigation strategies will result in the same crop response. In this case, a traditional agricultural DSS would probably recommend the irrigation strategy requiring least amount of water. Conversely, an ROI-based CPS framework would take into consideration additional factors including the costs associated with pumping energy for irrigation, the cost of water, expected increases in yield, and projected income. Thus, the selected action would represent not only physical/technical performance, but also financial viability. From a CPS viewpoint, both applications utilize the same general architecture: sensing supplies information about the current state of the system, ML predicts future states, a decision support layer assesses alternative choices using utility functions, and control modules implement the chosen course of action. Nonetheless, it is mainly the differing goals and objectives specific to each respective domain that define these applications, rather than any differences in the CPS architectural structure [13,17,25,44].

### 6.5. Uncertainty, Limited Data and Decision Quality

Uncertainty is met in both smart building and agriculture domains, yet in different manners. In smart buildings there is a high degree of uncertainty emerging from models used for simulation and occupant behavior. In agriculture there is unexpected environmental change and climate variability as well as inconsistent sensor data. As a result, across both domains, decision support architectures choose probabilistic, fuzzy or hybrid decision methods to address uncertainty and incomplete knowledge. Consequently, this further intensifies the need for decision-centric CPS designs which offer successful decisions. The uncertainties associated with real-world applications should be accounted for, as opposed to pursuing optimal results by assuming complete and accurate data [25,39,46].

It has been demonstrated through the literature in both domains that a high degree of accuracy in predictive models does not always equate to good decision outcomes. Rather, increasing emphasis appears on developing architectures that exhibit robustness, awareness of uncertainty, and tolerance to faults as main objectives [18,25,36,46].

### 6.6. Actuation and the “Last-Mile” Problem

The success of DSS in both smart buildings and precision agriculture is heavily dependent on actuation. Based on the coding summarized in Table S1, most included studies were classified as ML-as-support rather than ML-as-controller. This suggests that there are still many CPS DSS which remain focused on generating recommendations rather than making decisions autonomously. Only a few studies have identified that the “last mile” gap is caused by the lack of research into integration with control systems, system interconnectivity, and long-term validation on system performance [20].

### 6.7. Practical Transfer Opportunities Across Domains

Despite acknowledging the fact that there is a common CPS structure in smart buildings and precision agriculture is key for domain transferability, it is of high importance to illustrate design approaches that can be transferred across these domains and enhance decision support performance in both. Smart building DSS are generally more advanced in their exploitation of constrained optimization, supervisory control and explicit economic evaluation. This would provide useful insight into the design of agricultural CPS, where irrigation and resource allocation decisions are typically made using prediction models. The latter are seldom placed within explicit and structured (transparent) optimization frameworks which assess profitability, operating cost and resource tradeoffs.

On the other hand, precision agriculture presents significant design opportunities for smart buildings when dealing with sparsely populated, noisy, delayed or heterogeneously sensed deployments. Agricultural DSS are often designed to endure incomplete observations, unreliable communication (e.g., wireless connectivity) and environmental variability (e.g., weather). As such, there are several design approaches that could enhance the robustness of smart building CPS in deployment scenarios where sensor coverage is limited, building behavior is poorly modeled, or occupant reactions introduce high uncertainty. Examples include the usage of proxy variables, heuristic reasoning, uncertainty awareness and hybrid decision models.

Another potential practical benefit from this synthesis involves CPS performance evaluation. While both domains have developed many systems capable of generating recommendations for actions, few have achieved end-to-end integration with actuators. Therefore, an important methodological lesson learned through this cross-domain exploration is that future research on CPS should assess not only prediction accuracy but also decision quality, feasibility of decisions execution, robustness under uncertainty, and measurable outcomes after actuation. In essence, the cross-domain synthesis establishes a reusable set of design principles for decision support rather than a simple descriptive comparison between two application areas. These thoughts are depicted in Table 2.

### 6.8. Practical Deployment Limitations in Closed-Loop CPS DSS

While the literature examined in this paper commonly addresses sensing, prediction, decision making, and control in terms of conceptual CPS feedback loops, practical deployment limitations create barriers to effective decision support in actual environments. Decision quality is determined not only by predictive model design but also by communication performance, security, and reliability on the CPS overall [22,30,36].

A significant challenge is timing and communications latency. A closed-loop DSS expects that all information collected from sensors reaches the decision logic and any necessary actuators within an acceptable timeframe. In reality, delays can be introduced into the flow of information through intermittent or congested communications, cloud processing, and synchronization. These types of delays can negatively impact predictive control and ultimately result in decisions based upon outdated states of the system. The issue is especially relevant when considering distributed agricultural deployments and the growth pace of interconnected devices and subsystems in smart buildings. Consequently, future CPS architectures should evaluate not only prediction accuracy but also end-to-end decision timing and latency throughout the control loop [18,36,48].

Another challenge is related to scaling. Most experimental CPS designs are demonstrated using small sets of sensors, devices or physical objects. Conversely, operational deployments often include thousands of sensors, multiple and different communication platforms, and geographically distributed assets. As system size grows, so do the complexity of data management, computational needs and the cost associated with coordinating activities and model maintenance. Scaling impacts a CPS’ decision support capabilities to provide consistent levels of decision quality as new data or complexity are added. Various authors have recognized the necessity for scalable architectures that are able to support responsive and reliable large-scale distributed CPS [22,32].

A major concern is cybersecurity. Since the decision support layer strongly relies on the integrity and availability of sensed data, cyber-attacks such as false data injection, replay, Denial-of-Service (DoS) and disruption of communications can directly affect decision logic and subsequently lead to wrong actuations. The consequences of a failed CPS cybersecurity are further exacerbated if we reflect propagation to the physical world through improper control actions. Reviews have repeatedly emphasized that in order to achieve sufficient levels of resiliency against cyber threats, resilience should be treated as an integrated aspect of control systems design rather than merely treating it as a separate information technology function. Therefore, future CPS architectures should include mechanisms to enable secure communication, detect anomalies and authenticate/authorize users so that operations will not disrupt in the event of adverse operating conditions [58,59,60]. In combination, these findings show the path from promising laboratory prototypes into dependable real-world CPS implementations.

### 6.9. Effects on CPS Design and Research

The cross-domain comparisons provide some insight that can influence the design and development of DSS:

- Decision support should be considered a first-class CPS system component and be dissociated from the predictions’ degree of accuracy we get.
- Evaluation of ML capabilities must consider decision quality and control process outcomes, rather than just inference scoring.
- Economic objective functions still have an uneven role and non-explicit presence in many CPS applications, offering great potential for future CPS research.

These observations identify the CPS research opportunities and future work areas discussed in the final section. They are concentrated and compared in Table 3.

## 7. Future Research Opportunities

Many advances have been made in smart buildings and precision agriculture technologies. Our analysis, though, revealed areas that provide an opportunity for new research to develop more effective IoT and ML systems for decision support.

### 7.1. Decision Quality over Prediction Accuracy

We previously stated that considerable attention is given to predictive scores, leaving aside decision quality. Numerous reports analyze forecasting accuracy and its key importance. In contrast, few researchers study ways for forecasts to lead to better decisions. Smart buildings and precision agriculture future research will need to evaluate ML components in terms of decision effectiveness and real-world impacts without just staying at model metrics [14,18,39].

### 7.2. Economic Goals

The reductions in costs and ROI are important factors in decision making, but seldom are they incorporated into decision models. The building literature investigates economic benefits coming from energy savings after retrofit. Agricultural studies usually approach profits in an implicit manner. An interesting area of research would be the development of profit-oriented decision frameworks which can weigh economic criteria along with technical constraints [13,23,24].

### 7.3. Uncertainty and Limited Data

Both smart buildings and precision agriculture make decisions under uncertainty. The types of uncertainties differ between domains: uncertainty due to unknown occupancy behaviors and mismatched modeled responses in buildings; uncertainty in environmental variability and measurement imperfection existing in agriculture. Probabilistic/fuzzy decision support techniques have been reported in both domains; however, their application is disjointed. Therefore, future CPS research should concentrate on developing unified decision support architectures that are resilient to uncertainty and persist functionality regardless of data limitations, noise, and heterogeneity [18,39,48].

### 7.4. Linking Decision Logic and Actuation

As described in Section 6, the ‘last mile’ gap persists between decision generation and reliable execution. Many systems cease after providing recommendations on what actions to take. Thus, it is critical that future work emphasizes end-to-end CPS performance, which ensures decisions produced by ML systems are executed reliably, safely and consistently over time [18,36,39].

As discussed in Section 4.3 and Section 5.3, it is apparent that deep learning can deliver a successful means for mapping observed states into control actions, provided there is thoughtful incorporation of domain-specific knowledge, operational constraints and validation techniques. Thus, learning-based control in buildings and agricultural CPS should be seen as an evolving paradigm complementary to more widely accepted decision support/control practices.

### 7.5. Practical Evaluations

Empirical validations in both areas are restricted to simulations or short experimental runs. Studies about the long-term performance of DSS across their entire lifetime are rare as well as comparisons under realistic operating conditions. It is necessary to explore this area to understand how DSS behave over time, whether they adjust to varying conditions and if they produce sustainable financial benefits [25,39].

### 7.6. Cross-Domain Transfer and CPS Design Reuse

The similarities between smart buildings and precision agriculture identified in this study suggest that neither domain should be considered an isolated application silo. Instead, reusable CPS design patterns including utility-based decision support, uncertainty management, and closed-loop control integration may produce CPS frameworks for sharing methodology and knowledge across domains.

### 7.7. New Paradigms for AI and Next-Generation CPS Decision Making

In addition to the decision making issues that were identified in this survey of the literature, next-generation CPS will be impacted by new paradigms that are being developed in the AI space. The development of generative AI and foundational models provides promising approaches to systems that can combine information from different types of sensors (e.g., temperature, pressure, flow rate), historical records of system operation, relevant domain-specific knowledge, and human expertise into a common decision making framework. Unlike conventional ML models which are custom-tailored for a particular prediction problem, they can support multiple parallel functions including state estimation, forecasting, anomaly detection, explanation generation, and operator interactions. This capability is expected to result in more adaptable and more context-aware CPS architectures in both smart buildings and precision agriculture, where decision support that combines data from many different sources along with operational constraints and economic objectives is often required [61,62,63].

The development of digital twins constitutes yet another promising direction for future CPS evolution. Although most existing implementations of digital twins on CPS have focused primarily on monitoring and simulations, there exists the opportunity to integrate them with decision support logic to continuously evaluate possible control alternatives prior to their execution in the physical world. For example, digital twins in smart buildings might support optimizations of HVAC operations and energy management, while those for precision agriculture might support virtual testing of irrigation schedules, resource allocation policies, and climate adaptation strategies. Moreover, the ability to update digital representations of the CPS using real-time sensor data and ML inference poses the potential to improve decision making under uncertainty and to reduce operational risk [19,64].

Future CPS deployments are anticipated to leverage edge intelligence. Edge intelligence refers to occurrence of inference and decision support functions closer to the sensors and actuators rather than within centralized infrastructures. Edge-based architectures can reduce communication latency, improve reliability, and increase resiliency in areas characterized by unreliable or intermittent connectivity and strict real-time requirements. These attributes are especially relevant to agricultural deployments in remote areas and building control applications demanding rapid actuator response times [65].

However, as CPS grow autonomous via AI, numerous issues arise regarding trustworthiness, safety, transparency, explainability, and robustness. Controllers utilizing ML and generative decision support systems must exhibit reliable behavior over uncertain and dynamic operating conditions while being understandable to human operators. As a result, future research must focus on trustworthy AI mechanisms including uncertainty quantification, explainable decision support, cyber security protections, formally verified ML components, and human-in-the-loop governance structures [66]. Foundation models, digital twins, edge intelligence and trustworthy AI may provide the basis for a new generation of CPS decision support architectures that are adaptive, explainable, resilient and capable of supporting more complex operational goals.

## 8. Conclusions

This paper performed a review of decision support and control in CPS. This paper reviewed the literature by examining closed-loop CPS, IoT technology exploits in sensing, ML insertions points, and decision objectives and patterns.

Both fields showed us that CPS intelligent decision making requires more than just sensor accuracy and predictive capability. Decision making intelligence occurs when all elements (inference, decisions and actuation) are fully integrated into the closed-loop system. ML was also shown to enhance perception, prediction and evaluation. However, how the ML output is incorporated into a constrained domain-specific control process will determine whether reliable decision support will be generated. The coding results summarized in Table S1 and Figure 6 provide additional evidence that ML is mostly applied as a decision support tool and not as a full autonomous control mechanism.

A primary result of this study is that a robust CPS DSS is fundamentally based on logic and control rather than algorithmic excellence. The synthesis of the domains highlighted the distinctions between them and opportunities for improvement. While smart buildings pursue economic objectives and constrained optimization at a greater degree, precision agriculture has mechanisms to effectively operate under high environmental uncertainty and limited information.

This study emphasizes the need to evaluate CPS decision making based on decision quality, economic viability, and real-world impact and not solely upon predictive performance and technological innovation. Finally, this paper presents a unifying view from which design patterns may be generalized across various decision-intensive domains. Overall, this article establishes efficient decision support as a first-class concern within CPS research, while providing a guided foundation for future work on robust, economically viable and deployable CPS DSS in both built and natural environments.

## Figures and Tables

**Table 1:** Comparing previous articles to this review of ML based CPS decision support.

Articles of Representative Populations	Primary Goal	Decision Support and Control	Economic Goals	Synthesis Across Domains
Articles related to ML for energy utilization in smart buildings [11,12,13,14,15]	Prediction and optimization of energy use, and control methods in smart buildings	Partial	Secondary	No
Articles relevant to smart irrigation and precision agriculture [16,17,18,19,20,21]	IoT sensors for predicting water usage with ML, scheduling of irrigation systems	Partial	Usually implicit	No
This scoping review	Decision support and control in the CPS loops	Main focus	Explicit and systematic	Yes (building and agriculture)

**Table 2:** Transfer opportunities across domains.

Source Domain	Transferable Practice	Target Domain	Value
smart buildings	Constrained optimization/supervisory control	precision agriculture	more transparent irrigation/resource decisions
smart buildings	explicit ROI/cost-aware decision criteria	precision agriculture	better embedding of profitability into DSS logic
smart buildings	ROI-based retrofit decision frameworks	precision agriculture	irrigation scheduling based on expected economic return rather than water-use metrics alone
precision agriculture	robust operation under sparse/noisy sensing	smart buildings	more resilient control under limited observations
precision agriculture	hybrid reasoning (heuristics + ML + uncertainty handling)	smart buildings	better deployment robustness in real buildings
both	end-to-end evaluation from prediction to actuation	both	better assessment of operational effectiveness

**Table 3:** Observations across domains and research opportunities.

Dimension	Smart Buildings	Precision Agriculture	Cross Domain Insights—Transferability
CPS Structural Patterns (Feedback Loop)	Typical Closed-Loop Control Structure: Sensing → Predictions → Decisions → Actuations → Feedback	Similarly built on closed-loop sensing–analysis–decision–actuation cycles.	Both domains implement essentially the same control-centric CPS feedback loop. Decision support must be considered more as a control process than algorithm-centric.
ML Insertion Points & Roles	ML is primarily used to forecast and optimize building operations within established control frameworks. It serves as a predictive tool and does not replace decision logic.	ML is used at similar insertion points but often steps in for bad, noisy or missing data. It also serves as a predictive tool and does not replace decision logic.	Both domains integrate ML into their CPS loops for perception and prediction but with different emphases. Synergy across domains can be achieved by using smart buildings ML strength in control along with agriculture’s ML strength in bad/little data. Both domains can benefit from ML mechanisms which exploit measured data to directly produce actions.
Decision Objectives & Economic Goals	Mostly technical multi-objective (energy efficiency and occupant comfort). In some cases, explicit economic goals (energy cost savings, ROI in retrofit).	Multi-objective to balance water efficiency and crop yield. Economic factors (profit, cost) are mainly implicit or post hoc considerations not embedded directly in decision mechanisms.	Buildings’ strong control frameworks could enhance agricultural decision support, while agriculture’s horizontal approaches highlight the importance of embedding economic sustainability criteria more explicitly across both.
Uncertainty & Data Limitations	Uncertainty stems from unpredictable occupant behavior and model simplifications. High-volume sensor data.	Environmental and measurement uncertainties along with sensor data gaps are common. Measurements aggregation or heuristics are used to manage uncertainty.	Both domains can benefit from decision approaches resilient to uncertainty and incomplete data. The cross-domain insight is that CPS designs must prioritize efficient decisions under real-world constraints over result optimization under unrealistic data assumptions.
Decision Support vs. Actuation (Last Mile’)	Many smart buildings decision support solutions stop at recommendations, with few systems achieving full integration into automated building controls.	Similarly, many precision agriculture systems provide advice without closed-loop automated execution; integrating decision outputs with field actuators are ongoing challenges due to technical and environmental constraints.	Both domains face a “last mile” shortcoming where decisions must be translated into reliable actions. Cross-domain lesson is needed for end-to-end CPS implementations, ensuring that decision algorithms seamlessly drive actual controls.
Maturity & Deployment	Advanced CPS control paradigms (e.g., predictive control via IoT) have been demonstrated in buildings, but widespread deployment is limited by high upfront costs, integration complexity with legacy systems, and unclear ROI; many solutions remain at pilot or simulation stage rather than full commercial adoption.	Precision agriculture CPS have numerous prototypes and pilot deployments (e.g., IoT-based irrigation management), but broad adoption is tempered by connectivity/power constraints and farm heterogeneity; implementations are often limited to well-resourced or research contexts, with few mainstream, at-scale deployments yet.	Domain differences reflect context rather than any inherent maturity gap; each field has matured in certain aspects (buildings in formal optimization methods, agriculture in coping with harsh, distributed conditions). Cross-domain exchange of best practices (e.g., applying the building domain’s ROI-driven evaluation frameworks to agriculture, and adopting agriculture’s resilience techniques in building systems) could accelerate the practical deployment of CPS-based decision support in both domains.

## Data Availability

No new data were created or analyzed in this study. Data sharing is not applicable to this article.

## Supplementary Materials

The following supporting information can be downloaded at: https://www.mdpi.com/article/10.3390/s26144435/s1, Table S1: Characteristics of studies included in qualitative synthesis; Table S2: Taxonomy counts derived from Table S1; Table S1 presents the characteristics of the studies included in the qualitative synthesis, while Table S2 presents the taxonomy counts derived from Table S1. All references cited in Tables S1 and S2 are included in the main manuscript reference list.

## Abbreviations

The following abbreviations are used in this manuscript:

AI	Artificial Intelligence
CPS	Cyber–physical system
DSS	Decision support system
HVAC	Heating ventilation and air conditioning
IoT	Internet of Things
ML	Machine Learning
MPC	Model predictive control
ROI	Return on investment
