# Supplementary material for: Cyber-Physical System Integration of IoT Sensing and Machine Learning: A Cross-Domain Review of Decision Support and Control in Smart Buildings and Precision Agriculture
Source: Sensors (Basel). 2026 Jul 13;26(14):4435. doi: 10.3390/s26144435 (PMC13416872; doi:10.3390/s26144435)
Supplement: Supplementary file 1 [file sensors-26-04435-s001.zip › sensors-4415765-supplementary.pdf]

Table S1. Characteristics of studies included in qualitative synthesis. Note: Reference numbers correspond directly to the references list of the main manuscript.

Study Ref.	Domain	Type of Study	Primary ML Function	ML Decision/Control Role	Target	Evaluation setting
[11]	smart buildings	survey	ML applications for smart building energy utilization	ML-as-support	forecasting, energy use prediction, operational support	recommendation / indirect actuation
[12]	smart buildings	review	ML for smart and energy-efficient buildings	ML-as-support	energy managemen t and efficiency support	recommendation / indirect actuation
[13]	smart buildings	systematic review	AI for energy optimization in smart buildings	ML-as-support	energy optimizatio n and economic evaluation	recommendation / indirect actuation
[14]	smart buildings	scoping review	data-driven technologies for energy optimization	ML-as-support	energy decision support and deployment barriers	recommendation / indirect actuation
[15]	smart buildings	review	ML methods, challenges, and trends in smart buildings	ML-as-support	forecasting, diagnosis, building-managemen t support	recommendation / indirect actuation
[31]	smart buildings	review	smart building management systems as CPS applications	ML-as-support	monitoring and control support	mixed / semi-automated
[33]	smart buildings	control study	closed-loop identification for MPC of HVAC systems	ML-integrated supervisory control	predictive model support for HVAC control	closed-loop control
[34]	smart buildings	critical review	HVAC control-loop performance assessment	ML-integrated supervisory control	control-loop evaluation and performance assessment	closed-loop control
[35]	smart buildings	systematic review	IoT for energy management in smart buildings	ML-as-support	energy managemen t and	recommendation / indirect actuation

					deployment support	
[36]	smart buildings	systematic review	BEMS, sensors, IoT, and AI integration	ML-as-support	building energy managemen t and integration issues	mixed / semi-automated
[37]	smart buildings	review	AI evolution in smart buildings for energy efficiency	ML-as-support	energy optimizatio n and deployment support	recommendation / indirect actuation
[38]	smart buildings	review	MPC for smart buildings	ML-integrated supervisory control	MPC-based energy managemen t	closed loop / supervisory control
[39]	smart buildings	critical review	field implementations of MPC in built environments	ML-integrated supervisory control	predictive control under real deployment constraints	closed loop / supervisory control
[40]	smart buildings	experimental study	IoT-based hardware-in-the-loop MPC ventilation	ML-integrated supervisory control	ventilation control through MPC framework	closed loop control
[41]	smart buildings	research article	fast ML for building management systems	ML-integrated supervisory control	hybrid ML support for building managemen t optimizatio n	Supervisory control
[42]	smart buildings	simulation study	reinforcement learning vs. PID for HVAC control	ML-as-controller	learned HVAC control policy	closed loop control
[43]	smart buildings	experimental/simulati on study	reinforcement learning algorithms for building HVAC control	ML-as-controller	learned HVAC control strategies	closed loop control
[44]	smart buildings	control study	online identification and temperature tracking over IoT controller	ML-as-controller	learning-based temperature tracking control	closed loop control
[45]	smart buildings	case study	AI-enhanced techno-economic and environmental	ML-as-support	retrofit planning, economic/en vironmental	recommendation / planning

			optimization for building retrofit		decision support	
[46]	smart buildings	research article	AI temporal planning for energy smart buildings	ML-integrated supervisory control	planning and scheduling for coordinated energy decisions	semi-automated planning
[16]	precision agriculture	survey	ML-based DSS for smart irrigation scheduling	ML-as-support	irrigation scheduling support	recommendation / indirect actuation
[17]	precision agriculture	review	precision irrigation management using ML and digital farming	ML-as-support	irrigation decision support	recommendation / indirect actuation
[18]	precision agriculture	review	IoT-based automated solutions using ML for irrigation management	ML-as-support	irrigation managemen t support	mixed / semi-automated
[19]	precision agriculture	review	ML and digital twins in smart irrigation	ML-as-support	irrigation optimizatio n and scenario support	mixed / semi-automated
[20]	precision agriculture	review	ML-based precision agriculture techniques with IoT	ML-as-support	crop monitoring and farming decision support	recommendation / indirect actuation
[21]	precision agriculture	systematic review	ML and big data in intelligent agricultural ecosystems	ML-as-support	agricultural ecosystem DSS	recommendation / indirect actuation
[23]	precision agriculture	systematic review	drivers and barriers for Agriculture 4.0	ML-as-support	adoption and decision-support context	recommendation / indirect actuation
[24]	precision agriculture	survey	big data and AI in precision agriculture	ML-as-support	resource-managemen t and analytics support	recommendation / indirect actuation
[25]	precision agriculture	systematic review	CPS for smart farming	ML-as-support	smart farming DSS and CPS integration	recommendation / indirect actuation

[49]	precision agriculture	review	smart sensors and IoT in precision agriculture	ML-as-support	sensing and monitoring support for decisions	recommendation / indirect actuation
[50]	precision agriculture	systematic review	digitization and Agriculture 4.0	ML-as-support	digital transformation and resource-management support	recommendation / indirect actuation
[51]	precision agriculture	research article	crop model and improved deep reinforcement learning for irrigation scheduling	ML-as-controller	learned irrigation scheduling policy	closed loop / policy-based control
[52]	precision agriculture	research article	reinforcement-learning-based intelligent irrigation decision-making model	ML-as-controller	learned irrigation decision policy	closed loop / policy-based control
[53]	precision agriculture	research article	deep reinforcement learning for irrigation efficiency in the field	ML-as-controller	learned irrigation control	closed loop / policy-based control
[54]	precision agriculture	research article	reinforcement learning for greenhouse irrigation control	ML-as-controller	learned greenhouse irrigation control	closed loop control
[55]	precision agriculture	research article	deep reinforcement learning for irrigation scheduling with sensor feedback	ML-as-controller	learned irrigation scheduling policy	closed loop / policy-based control
[56]	precision agriculture	book chapter / review	DSS in precision agriculture and conservation	ML-as-support	precision agriculture decision support	recommendation / indirect actuation
[57]	precision agriculture	research article	Computational approaches for tree-crop irrigation decision support	ML-as-support	irrigation DSS under uncertainty	recommendation / decision support
[58]	precision agriculture	review	DSS in precision farming	ML-as-support	precision farming decision support	recommendation / indirect actuation

Table S2. Taxonomy counts derived from Table S1

Studies Ref.	ML Decision/Control Role	Count	Percentage
[11–15], [31], [35–37], [45], [16–21], [23–25], [49], [50], [56–58]	ML-as-support	24	61.5
[33], [34], [38–41], [46]	ML-integrated supervisory control	7	18
[42–44], [51–55]	ML-as-controller	8	20.5
Total	---	39	100
